# A Resistant Case of Familial Nonsexual Transmission of Trichomonas

**DOI:** 10.7759/cureus.9246

**Published:** 2020-07-17

**Authors:** Kelley M Bishop

**Affiliations:** 1 Family Medicine, University of Mississippi Medical Center, Jackson, USA

**Keywords:** trichomonas, resistant, nonsexual

## Abstract

Trichomonas vaginalis is a protozoan responsible for the most prevalent sexually transmitted nonviral disease worldwide. This is a case report of probable grandmother to granddaughter nonsexual transmission of trichomonas. The granddaughter had no prior sexual contact for the last 11 years.

The patient presented for a follow-up of urinary tract infection diagnosed at her neurologist’s office. On review, it was noticed that the urinalysis microscopy revealed trichomonas. History-taking revealed no usual sexual exposure, but subsequent investigation revealed probable exposure from her grandmother. Furthermore, typical single-dose treatment followed by typical seven-day treatment was ineffective. High-dose metronidazole for seven days was required for treatment.

While nonsexual transmission of trichomonas is rare, there have been numerous case reports and studies showing that it does occur. The subsequent presented case highlights the need to broaden the search for contacts beyond sexual transmission. Also, resistance to usual treatment, as seen in this case, is seldom encountered, but it could have been caused by repeated treatment and re-exposure to the unusual source.

## Introduction

Trichomoniasis is a parasitic infection typically of the genitourinary system that has significance as being one of the most common and the most curable sexually transmitted infections worldwide [1]. While the disease is most commonly transmitted through sexual contact, there have been case reports of nonsexual transmission [2]. Survival of trichomonas on fomites has been reported, but no direct link to transmission has been made [3,4].

Randomized clinical trials using single-dose regimens of metronidazole have shown 84-98% cure rates [5-7]. Resistant cases of trichomoniasis to metronidazole occur in only 4-10% of cases [8,9]. Because of this low resistance, the possibility of re-infection must be distinguished. Re-infection has been seen to occur at a high rate of up to 17 % within three months [10].

## Case presentation

A 26-year-old black female with a history of a chronic autoimmune disorder presented for follow up of urinary tract infection (UTI) diagnosed at a neurology appointment. While she had been given appropriate treatment for the UTI, the incidental finding of trichomonas on urinalysis microscopy had been overlooked. The patient denied having sexual activity for over 10 years. She was asymptomatic, having no vaginal discharge or irritation. A wet prep was done at the visit, which again showed trichomonas. On chart review, it was found that she had been diagnosed with trichomonas four years prior incidentally by Pap smear and treated with 2 grams of metronidazole. Also seen on chart review was another urine microscopy positive for trichomonas six months prior to current presentation, again treated with 2 grams of oral metronidazole. The patient reported having urinary discomfort at that presentation. She stated that she had been compliant with both treatments. A decision was made to treat with metronidazole 500 mg twice daily for seven days. The patient gave permission to discuss the results and her diagnosis with her grandmother, who is the only person she lives with. Her grandmother then came into the clinic and was tested for trichomonas by wet prep, which returned positive results. Discussion revealed that the grandmother had not had sexual activity for the last three years, the last being with her now deceased husband. She was asymptomatic, denying vaginal discharge or irritation. She had not had a Pap smear, wet prep, or urine microscopy in over five years. On query, the patient and the grandmother stated that they did share a bathroom and bathtub. They both reported that they mostly took showers, only rarely a tub bath. They denied sharing washcloths or towels. They have separate soaps, but the patient admitted that she had on a rare occasion used her grandmother’s soap. They vehemently denied the use of sex toys or of sharing of such. The grandmother was given metronidazole 500 mg twice a day for seven days. Upon retesting by DNA polymerase chain reaction (PCR) after treatment, the grandmother was negative for trichomonas. The patient (granddaughter) returned three weeks later after treatment for retesting. At that time, DNA PCR testing was performed, which revealed positive results again. A higher dosing of metronidazole at 1,000 mg twice daily for seven days was given at that time. Subsequent retesting for test of cure with trichomonas DNA PCR testing was negative.

## Discussion

According to the CDC (Centers for Disease Control and Prevention), approximately 3.7 million people in the United States suffer from trichomoniasis. The prevalence of trichomonas in reproductive-aged women was estimated by the National Health and Nutrition Examination Survey, where 3,754 women aged 14 to 49 years performed self-collected vaginal swabs, which were subsequently tested by PCR for trichomonas. The prevalence in this study was shown to be 3.1%, with increasing prevalence with age. Within that survey, the highest prevalence was seen at 13.3% in non-Hispanic black women and the lowest prevalence at 1.3% in non-Hispanic white women [11].

While the usual mode of transmission has been seen as sexual, nonsexual transmission has been described [2]. A report submitted as a letter to the editor gave evidence of transmission from a mother to two of her small children [12]. A study conducted in Zambia in 2011 of adolescent girls showed an incidence of approximately 25% who had never been sexually active. Inconsistent use of soap was seen as a statistically significant risk factor [13]. A 1991 communication discussed an epidemic of trichomoniasis in young girls in a remote Indian village, suggesting transmission through contaminated water [14]. In our case, a possible transmission from grandmother to granddaughter occurred through the rare sharing of bathcloths/sponges/bath soap. While the grandmother had possible exposure through prior sexual activity with her late husband, the granddaughter denied any sexual activity in the recent past. Of note, they also both denied usage of sex toys. One published study showed viability of most isolates of trichomonas on nonabsorbent material and almost half of isolates on adsorbent material at four hours, with one isolate showing viability up to 24 hours on nonabsorbent material [4].

As stated earlier, cure rates of trichomonas with metronidazole have been seen to approach 85-90%. In one study, treatment groups using a single 2-gram dose achieved an 86% cure rate, whereas groups using standard seven-day treatment of 250 mg three times a day achieved a 91.6% cure rate [15]. Treatment failure rates were seen to be higher in the single-dose regimen in a 2017 meta-analysis [16]. In a surveillance study published in 2012 of 538 Trichomonas vaginalis isolates, none exhibited moderate- to high-level metronidazole resistance [8]. This was borne out in the presented case in that the granddaughter had shown a resistance not only to the single-dose regimen but also to a standard seven-day regimen. She required a higher dose of metronidazole regimen to achieve a cure.

## Conclusions

While the typical case of trichomonas can be simply traced to sexual exposure, this case demonstrates that atypical nonsexual exposure can be seen, albeit rarely. Thorough history-taking and curiosity revealed that the granddaughter’s only probable exposure was a non-traditional source from her grandmother. She was likely re-infected after past treatments through this exposure. The cycle of treatment and re-exposure almost assuredly contributed to the resistant strain of trichomonas. Diligence in searching out her exposure source should prevent re-infection from that source and has led to her treatment.

## References

[1] Petrin D, Delgaty K, Bhatt R, Garber G (1998). Clinical and microbiological aspects of Trichomonas vaginalis. Clin Microbiol Rev.

[2] Kandamuthan S, Thambi R, Yesodharan J (2014). Trichomoniasis: is it always sexually transmitted?. Indian J Sex Transm Dis AIDS.

[3] Whittington M (1957). Epidemiology of infections with Trichomonas vaginalis in the light of improved diagnostic methods. Brit J Vener Dis.

[4] Casero R (2015). Survival of Trichomonas Vaginalis Outside the Human Body and the Effect of Biocides in Killing Parasites In Vitro.

[5] Spence MR, Harwell TS, Davies MC (1997). The minimum single oral metronidazole dose for treating trichomoniasis: a randomized, blinded study. Obstet Gynecol.

[6] Gabriel G, Robertson E, Thin RN (1982). Single dose treatment of trichomoniasis. J Int Med Res.

[7] Thin RN, Symonds MA, Booker R, Cook S, Langlet F (1979). Double-blind comparison of a single dose and a five-day course of metronidazole in the treatment of trichomoniasis. Br J Vener Dis.

[8] Kirkcaldy RD, Augostini P, Asbel LE (2012). Trichomonas vaginalis antimicrobial drug resistance in 6 US cities, STD Surveillance Network, 2009-2010. Emerg Infect Dis.

[9] Schwebke JR, Barrientes FJ (2006). Prevalence of Trichomonas vaginalis isolates with resistance to metronidazole and tinidazole. Antimicrob Agents Chemother.

[10] Peterman TA, Tian LH, Metcalf CA (2006). High incidence of new sexually transmitted infections in the year following a sexually transmitted infection: a case for rescreening. Ann Intern Med.

[11] Sutton M, Sternberg M, Koumans EH, McQuillan G, Berman S, Markowitz L (2007). The prevalence of Trichomonas vaginalis infection among reproductive-age women in the United States, 2001-2004. Clin Infect Dis.

[12] Adu-Sarkodie Y (1995). Trichomonas vaginalis transmission in a family. Genitourin Med.

[13] Crucitti T, Jespers V, Mulenga C, Khondowe S, Vandepitte J, Buve A (2011). Non-sexual transmission of trichomonas vaginalis in adolescent girls attending school in Ndola, Zambia. PLoS ONE.

[14] Charles SX (1991). Epidemiology of trichomonas vaginalis (TV) in rural adolescent and juvenile children. J Trop Pediatr.

[15] Hager WD, Brown ST, Kraus SJ, Kleris GS, Perkins GJ, Henderson M (1980). Metronidazole for vaginal trichomoniasis: seven-day vs single-dose regimens. JAMA.

[16] Howe K (2017). Single-dose compared to multi-dose metronidazole for the treatment of trichomoniasis in women: a meta-analysis. Sex Transm Dis.

